# Fabrication of Metastable Crystalline Nanocomposites by Flash Annealing of Cu_47.5_Zr_47.5_Al_5_ Metallic Glass Using Joule Heating

**DOI:** 10.3390/nano10010084

**Published:** 2020-01-01

**Authors:** Ilya Okulov, Ivan Soldatov, Ivan Kaban, Baran Sarac, Florian Spieckermann, Jürgen Eckert

**Affiliations:** 1 Faculty of Production Engineering, University of Bremen, Badgasteiner Street 1, 2, 28359 Bremen, Germany; 2 Leibniz Institute for Materials Engineering—IWT, Badgasteiner Street 3, 28359 Bremen, Germany; 3 Leibniz Institute for Solid State and Materials Research IFW Dresden, Helmholtzstrasse 20, 01069 Dresden, Germany; i.soldatov@ifw-dresden.de (I.S.); i.kaban@ifw-dresden.de (I.K.); 4 Institute of Natural Sciences and Mathematics, Ural Federal University, 620002 Yekaterinburg, Russia; 5 Erich Schmid Institute of Materials Science, Austrian Academy of Sciences, Jahnstraße 12, 8700 Leoben, Austria; baran.sarac@oeaw.ac.at (B.S.); juergen.eckert@unileoben.ac.at (J.E.); 6 Department of Materials Science, University of Leoben, Jahnstraße 12, 8700 Leoben, Austria; florian.spieckermann@unileoben.ac.at

**Keywords:** nanocomposite, metallic glass, flash annealing, metastable material, mechanical behaviour

## Abstract

Flash Joule-heating was applied to the Cu_47.5_Zr_47.5_Al_5_ metallic glass for designing fully crystalline metastable nanocomposites consisting of the metastable B2 CuZr and low-temperature equilibrium Cu_10_Zr_7_ phases. The onset of crystallization was in situ controlled by monitoring resistivity changes in the samples. The effect of heating rate and annealing time on the volume fraction of the crystalline phases and mechanical properties of the nanocomposites was studied in detail. Particularly, an increase of the heating rate and a decrease of the annealing time lead to a lower number of equilibrium Cu_10_Zr_7_ precipitates and an increase of tensile ductility. Tailoring of these non-equilibrium microstructures and mechanical properties may not be possible unless one starts with a fully glassy material that opens new perspectives for designing metastable nanomaterials with unique physical properties.

## 1. Introduction

For several decades, composite materials [1,2,3,4,5,6] and materials with composite microstructures [7,8,9] have been the focus of research and industry as they provide a whole range of complementary physical properties determined by matrix and non-matrix counterparts. In many cases, the properties of composite materials even exceed the properties of their constituent materials. Some examples include anomalously low elastic modulus in Fe-Mg microcomposites [10], outstanding strength in metal-polymer nanocomposites [5,11,12,13] fabricated from nanoporous metals [14,15,16,17,18,19], and significantly enhanced plastic deformability in metallic glass composites [20,21]. The design of non-equilibrium composite microstructures in as-cast nanostructured titanium alloys [22,23,24,25,26] leads to the high strength and good plastic deformability required for structural and biomedical applications [27,28]. One of the promising processing methods for the design of advanced materials with non-equilibrium composite microstructures is the annealing of metallic glasses [21,29].

Metallic glasses are metallic solids with disordered, liquid-like atomic structures. Thermodynamically, metallic glasses are in a high-energy (metastable) state regardless of their fabrication method and, consequently, they can be easily transformed into more stable crystalline states by lowering their energy. Among the exciting applications making use of annealing of metallic glasses is the development of nanostructured FINEMET material [8] with superior magnetic properties. Some further examples of composite-structured materials based on metallic glasses can be found in [30,31,32]. It is important to note that, usually, phase transformations in metallic glasses occur rapidly and, thus, rapid annealing methods have to be used in order to control these transformations.

Recently, flash-annealing techniques based on Joule [20], inductive [33,34], and electromagnetic radiation [35] heating were reported as tools for controllable tuning of the microstructure in metallic glass ribbons and bulk samples, respectively, aiming to improve their mechanical properties. Both techniques were applied to CuZr-based metallic glasses, which were partially devitrified into the metastable B2 CuZr structure to form glass-matrix composites. The thus obtained homogeneously distributed B2 CuZr crystals in the glassy matrix result in a significant improvement of the mechanical properties of the CuZr-based metallic glasses and even allow for tailoring the tensile ductility [20]. Furthermore, it has been shown that the strength of fully crystalline composites containing ultrafine-grained crystalline phases even exceeds that of the parent glass [20].

In this study, we focus on the effect of heating rate and time on the formation of stable and metastable crystalline phases in the Cu_47.5_Zr_47.5_Al_5_ metallic glass upon flash annealing and the mechanical properties of the crystalline nano- and microcomposites obtained.

## 2. Materials and Methods

Samples were prepared under high purity argon atmosphere in two steps. First, Cu_47.5_Zr_47.5_Al_5_ (at. %) ingots were produced from Cu (99.99%), Zr (99.98%), and Al (99.99%), by arc-melting. In the second step, glassy ribbons were prepared from the ingots by melt-spinning. The metallic glass ribbons were annealed using an in-house designed set-up. The samples were characterized by X-ray diffraction (XRD, Stoe, STADI P with Mo-Kα1 radiation, Darmstadt, Germany) and scanning electron microscopy (SEM, Zeiss, Leo Gemini 1530, Oberkochen, Germany). Phase identification was done by means of the X’Pert High Score Plus (Malvern Panalytical, Malvern, UK) software, whereas SEM images were analysed using ImageJ (open source) software. Mechanical testing was performed with an Instron 8562 machine (Instron, Norwood, MA, United States) at a strain rate of 10^−4^ s^−1^ at room temperature. The strain was measured by a laser extensometer (Fiedler Optoelektronik, Lützen, Germany). The gauge length of 5 mm was selected in the middle region of a ribbon sample.

## 3. Results and Discussion

**Figure 1:**
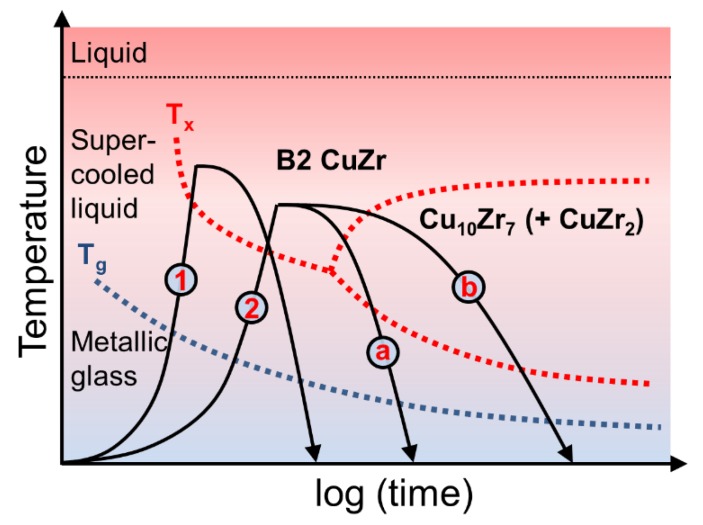
Schematic continuous heating transformation (CHT) diagram of the Cu_47.5_Zr_47.5_Al_5_ metallic glass based on [20].

Cu_47.5_Zr_47.5_Al_5_ metallic glass can transform to a lower energy state through several devitrification paths. These are schematically illustrated in a continuous heating transformation (CHT) diagram in Figure 1.

Fast heating to temperatures above the crystallization temperature, T_x_, as depicted by line 1, leads to nucleation of the metastable B2 CuZr phase in the metallic glass matrix. Fast cooling is required to avoid decomposition of B2 CuZr into the thermodynamically favourable low temperature eutectic phases (LT-EPs) Cu_10_Zr_7_ and CuZr_2_, and thus to stabilize B2 CuZr at room temperature. The ductile B2 CuZr crystals hinder localization of deformation in the glassy phase leading to tensile ductility of the glass-matrix composites [20]. Moving the cooling curve closer to the nose of the stability regime of the LT-EPs leads to a higher solid fraction of B2 CuZr, ultimately leading to complete suppression of the glassy phase. Heating along line 2 and cooling along lines a and b results in devitrification of the metallic glass into B2 CuZr with subsequent decomposition of B2 CuZr into the LT-EPs. As it has been shown in earlier works [20,33], the mechanical properties of the composites obtained depend strongly on the constituent phases.

In this work, we use the Joule heating for annealing of the Cu_47.5_Zr_47.5_Al_5_ metallic glass. Hereby, an electrical current is applied to a sample for a short time from several milliseconds to a few seconds, as described elsewhere [20]. The fast heating is favoured by the rather high resistivity of metallic glasses [36,37]. Since crystalline phases possess a higher conductivity compared to their glassy counterparts, devitrification of the glassy phase leads to a remarkable resistivity drop, which allows one to quite sensitively monitor crystallization processes [20]. The distribution of the applied current density versus the onset of the resistivity drop for the Cu_47.5_Zr_47.5_Al_5_ metallic glass can be found in our previous study [20]. The relatively short heating time during the Joule heating allows the adiabatic conditions to be nearly fulfilled and, therefore, the current density is proportional to the heating rate, as has been proven by measurement of heating rates using a thermocouple. The heating rates given in Table 1 have been estimated based on the crystallization temperature of the Cu_47.5_Zr_47.5_Al_5_ metallic glass (about 695 K at about 0.7 K s^−1^ heating rate) [38], and the measured time-to-crystallisation indicated by the resistivity drop. Particularly, the heating rate corresponding to the highest applied current density (namely, 59 ± 5 MA m^−2^) is ≥830 K s^−1^.

**Figure 2:**
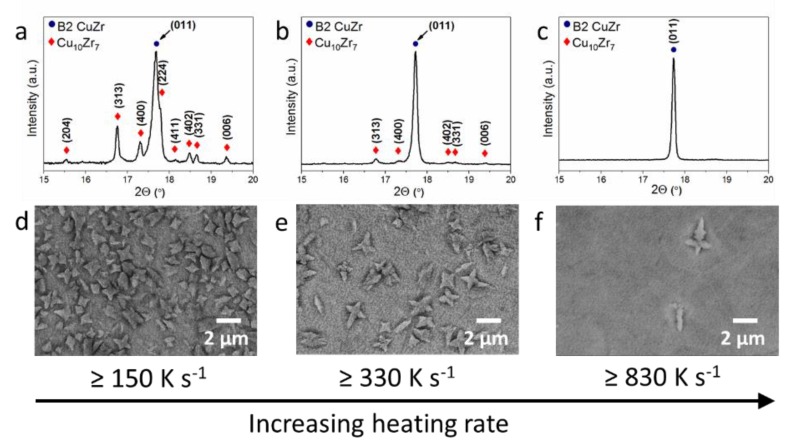
Effect of heating rate on the microstructure of flash annealed Cu_47.5_Zr_47.5_Al_5_ metallic glass. XRD patterns (**a**–**c**) and secondary electron SEM micrographs (**d**–**f**) of the Cu_47.5_Zr_47.5_Al_5_ metallic glass samples rapidly annealed by Joule heating (up to the resistivity drop) with the following current densities: (**a**,**d**) 34 ± 5 MA m^−2^; (**b**,**e**) 44 ± 5 MA m^−2^; and (**c**,**f**) and 59 ± 5 MA m^−2^. (Figure 2d is adopted from [20]).

Figure 2 displays the effect of the heating rate (current density) on the microstructure of the flash-annealed Cu_47.5_Zr_47.5_Al_5_ metallic glass. X-ray diffraction analysis of the samples annealed at the lowest heating rate (≥150 K s^−1^) reveals the presence of two crystalline phases: the low-temperature equilibrium Cu_10_Zr_7_ and the metastable B2 CuZr phases (Figure 2a). As the heating rate increases above 330 K s^−1^, the intensity of Cu_10_Zr_7_ reflections decreases significantly, indicating a lower content of this phase in the sample (Figure 2b). Finally, at a heating rate of ≥830 K s^−1^, there are no detectable peaks of the Cu_10_Zr_7_ phase and the sample mainly consists of the metastable B2 CuZr phase (Figure 2c).

Figure 2d–f demonstrates the microstructure of the annealed Cu_47.5_Zr_47.5_Al_5_ metallic glass samples. The samples consist of dendritic Cu_10_Zr_7_ crystals that are homogeneously distributed in a B2 CuZr matrix. The volume fraction of the Cu_10_Zr_7_ dendrites increases with decreasing heating rate from about 2 ± 1 vol.% (≥830 K s^−1^) to 17 ± 3 vol.% (≥330 K s^−1^), finally reaching 41 ± 5 vol.% at the lowest heating rate (≥150 K s^−1^) (Table 1). The size of the Cu_10_Zr_7_ dendrites also depends on the applied heating rate or current density. A larger dendrite size is achieved at a higher heating rate and vice versa. For example, the mean dendrite size in the sample subjected to the highest heating rate ≥830 K s^−1^ is 2.3 ± 0.2 µm, while it drops to 1.1 ± 0.3 µm for the samples obtained at the heating rate ≥150 K s^−1^ (Table 1). Along with the volume fraction and size of the Cu_10_Zr_7_ crystals, their number increases with higher heating rate from about 1.1 × 10^4^ to 22.0 × 10^4^ and reaches 61.2 × 10^4^ particles per mm^2^.

**Figure 3:**
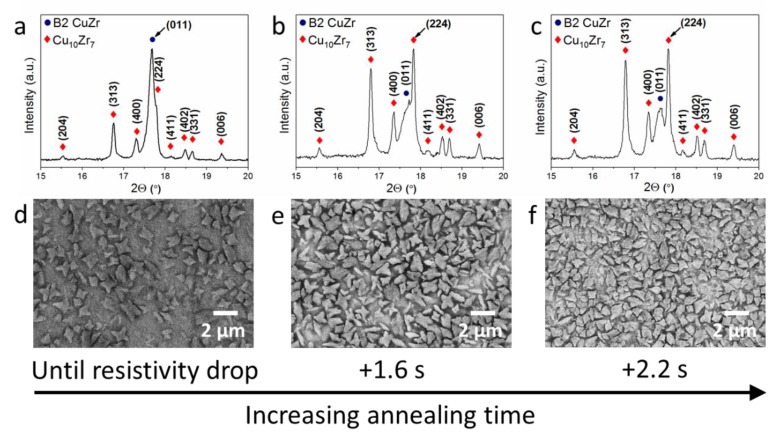
Effect of annealing time on the microstructure of flash annealed Cu_47.5_Zr_47.5_Al_5_ metallic glass. X-ray diffractograms (**a**–**c**) and secondary electron micrographs (**d**–**f**) of the Cu_47.5_Zr_47.5_Al_5_ metallic glass samples rapidly annealed by Joule heating at the current density *i*_3_ = 34 ± 5 MA m^−2^ and different times: (**a**,**d**) until the resistivity drop; (**b**,**e**) 1.6 s after the resistivity drop; and (**c**,**f**) 2.2 s after the resistivity drop. (Figure 3d is adopted from [20]).

The volume fraction of Cu_10_Zr_7_ dendrites in the B2 CuZr matrix can also be tuned by controlling the annealing time at a constant current density (heating rate), as shown in Figure 3. To reveal this effect, several samples annealed (i) until the resistivity drop, (ii) 1.6 s after the resistivity drop, and (iii) 2.2 s after the resistivity drop at a current density *i*_3_ = 34 ± 5 MA m^−2^ (≥150 K s^−1^) were selected. X-ray analysis indicates the presence of two phases in these samples, namely, the low-temperature equilibrium Cu_10_Zr_7_ phase and the metastable B2 CuZr phase. The intensity of the B2 CuZr phase peaks is highest for the samples annealed until the resistivity drop. A relatively small increase in annealing time (1.6–2.2 s) leads to a higher intensity of the Cu_10_Zr_7_ peaks. This is in agreement with the findings from secondary electron micrographs (Figure 3d–e). The increase of annealing time at the constant heating rate (≥150 K s^−1^) leads to a higher volume fraction and a larger number of Cu_10_Zr_7_ dendrites (Table 1). Particularly, the volume fraction of the Cu_10_Zr_7_ dendrites increases from 41 ± 5 to 89 ± 3 vol.% for the annealing times until the resistivity drop and 2.2 s after the drop, respectively. In contrast to the effect of heating rate, the size of the Cu_10_Zr_7_ dendrites varies insignificantly for different annealing times. These findings suggest that the volume fraction, number, and size of the Cu_10_Zr_7_ crystals strongly depend on the specific heat treatment conditions and can be tuned by varying the current density being proportional to the heating rate.

In the current study, the flash Joule heating of the Cu_47.5_Zr_47.5_Al_5_ metallic glass leads to its devitrification into two phases: metastable B2 CuZr and equilibrium Cu_10_Zr_7_. This finding is in contrast to the equilibrium phase diagram [39] and some experimental as well as theoretical studies on the devitrification sequence of CuZr-based metallic glasses [33,35,38,40]. Based on theoretical considerations, Kaban et al. suggested that the Cu_47.5_Zr_47.5_Al_5_ metallic glass devitrifies following the sequence Cu_10_Zr_7_→CuZr_2_→B2 CuZr [40]. Experimental studies show that the Cu_47.5_Zr_47.5_Al_5_ metallic glass directly transforms into the Cu_10_Zr_7_ and CuZr_2_ equilibrium phases upon annealing at low heating rates of 10–40 K min^−1^, while the CuZr_2_ phase precipitates after the Cu_10_Zr_7_ phase [38]. Recent reports demonstrated that rapid annealing of the Cu_47.5_Zr_47.5_Al_5_ metallic glass can suppress the formation of the low-temperature equilibrium phases completely [33] or partially [20], leading to its transformation into the non-equilibrium B2 CuZr phase. In the latter case, the Cu_10_Zr_7_ crystals are found within the B2 CuZr precipitates but the second equilibrium CuZr_2_ phase is not observed. According to the devitrification experiments and theoretical studies [38,40], the CuZr_2_ phase precipitates after Cu_10_Zr_7_, and, therefore, it can be assumed that in the current flash annealing case, the conditions for the precipitation of the CuZr_2_ phase are not fulfilled. The CuZr_2_ phase requires a specific stoichiometry for nucleation, which is probably not achieved during the short processing time.

**Figure 4:**
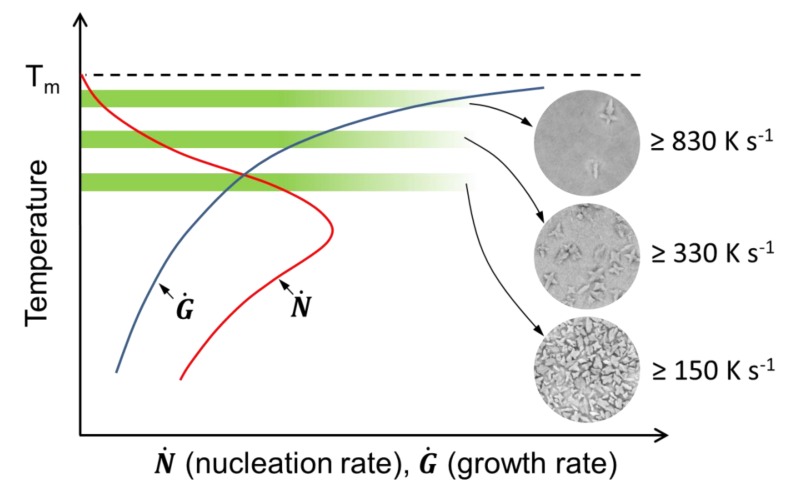
Schematic illustration of annealing conditions of the samples selected for the current study. Nucleation and growth rate curves are drawn based on reference [41].

The annealing conditions such as annealing temperature and time affect the nucleation and growth rate of precipitates. The growth rate is determined by the rate of diffusion and, therefore, it increases with increasing temperature [41]. The nucleation rate is also temperature-dependent and exhibits a maximum in an intermediate temperature range (Figure 4). According to the obtained results, the number of Cu_10_Zr_7_ dendrites decreases while their average size increases at higher heating rate. This suggests that the B2-98 sample with the lowest number (1.1 × 10^4^ ± 0.1 × mm^−2^) and the largest size (2.3 ± 0.3 µm) of Cu_10_Zr_7_ dendrites was subjected to the highest transformation temperature. A decrease of the heating rate leads to a larger number of Cu_10_Zr_7_ dendrites, which also become finer. This can be explained by a decrease of the average transformation temperature, which seems to be dependent on heating rate. The lowest applied heating rate (here, 150 K s^−1^) corresponds to the most optimum average transformation temperature for the highest nucleation rate. Therefore, increasing the annealing time in this case leads to a significant increase of the number of Cu_10_Zr_7_ dendrites.

**Figure 5:**
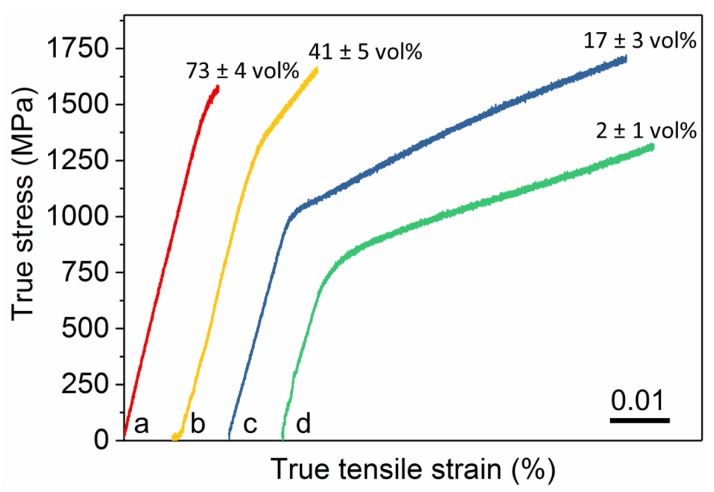
Room temperature tensile true stress-true strain curves of the Cu_10_Zr_7_ dendrite/B2 nano- and microcomposites obtained by flash annealing the Cu_47.5_Zr_47.5_Al_5_ metallic glass. (a) Annealed at 34 ± 5 MA m^−2^ until 1.6 s after the onset of the resistivity drop; (b) annealed at 34 ± 5 MA m^−2^ until the resistivity drop; (c) annealed at 44 ± 5 MA m^−2^ until the resistivity drop; and (d) annealed at 59 ± 5 MA m^−2^ until resistivity drop. The values at the end of the stress-strain curves indicate the volume fraction of the Cu_10_Zr_7_ dendrites. (Stress-strain curve “d” is adopted from [20]).

Tailored by flash annealing of the Cu_47.5_Zr_47.5_Al_5_ metallic glass, the composite microstructure of the current samples leads to notable tensile deformability and high strength (Figure 5) comparable with that of metallic glass matrix composites [20,33]. Moreover, the crystalline samples exhibit pronounced strain-hardening behaviour. The yield strength of the samples increases from 700 ± 30 to 1440 ± 30 MPa with increasing volume fraction of Cu_10_Zr_7_ dendrites (Table 2). This strength increase with increasing volume fraction of Cu_10_Zr_7_ dendrites is at the cost of tensile deformability: the strain-to-fracture decreases form 7.1 ± 0.5 to 1.8 ± 0.2% when the volume fraction of Cu_10_Zr_7_ dendrites increases from 2 ± 1 to 73 ± 4 vol.%. However, these fracture strain values are still in the range of interest for technological applications. For example, the B2-83 sample exhibits a tensile ductility of 7.5 ± 0.5%. Due to increasing strain hardening, the ultimate tensile strength increases from 1580 ± 50 to 1710 ± 50 MPa with higher volume fraction and number of Cu_10_Zr_7_ dendrites in the B2 CuZr matrix (Table 2).

## 4. Conclusions

In summary, we have tailored different microstructures by devitrification of the Cu_47.5_Zr_47.5_Al_5_ metallic glass using flash Joule annealing. The size and volume fraction of the constituent phases—metastable B2 CuZr and equilibrium Cu_10_Zr_7_—can be flexibly tuned by optimizing the heating rate and annealing time. The strength of the Cu_10_Zr_7_/B2 nanocomposites obtained through flash Joule heating and annealing exceeds that of the initial metallic glass and is comparable with that of metallic glass matrix composites. Hence, glassy materials provide a unique base for obtaining non-equilibrium microstructures by flash annealing with technologically attractive properties that cannot be achieved through conventional processing.

## Figures and Tables

**Table 1:** Microstructural characteristics of the Cu_47.5_Zr_47.5_Al_5_ metallic glass samples rapidly annealed until the resistivity drop.

Sample	Current Density (MA m^−2^)	Estimated Heating Rate (K s^−1^)	Annealing Time	Volume Fraction of B2 CuZr (vol.%)	Volume Fraction of Cu_10_Zr_7_ (vol.%)	Number of Cu_10_Zr_7_ Particles (mm^−2^)	Size of Cu_10_Zr_7_ Particles (µm)
B2-98	59 ± 5	≥830	Until resistivity drop	98 ± 1	2 ± 1	1.1 × 10^4^ ± 0.1	2.3 ± 0.3
B2-83	44 ± 5	≥330		83 ± 3	17 ± 3	22.0 × 10^4^ ± 0.7	1.7 ± 0.2
B2-59	34 ± 5	≥150		59 ± 5	41 ± 5	61.2 × 10^4^ ± 1.5	1.1 ± 0.3
B2-27	34 ± 5	≥150	1.6 s after resistivity drop	27 ± 4	73 ± 4	146.9 × 10^4^ ± 2.8	1.2 ± 0.2
B2-11	34 ± 5	≥150	2.2 s after resistivity drop	11 ± 3	89 ± 3	169.5 × 10^4^ ± 2.1	1.0 ± 0.2

**Table 2:** Mechanical properties of the Cu_10_Zr_7_ dendrite/B2 nano- and microcomposites obtained by flash annealing the Cu_47.5_Zr_47.5_Al_5_ metallic glass.

Sample	Yield Strength (MPa)	Ultimate Tensile Strength (MPa)	Young’s Modulus (GPa)	Strain to Fracture (%)
B2-27	1440 ± 30	1580 ± 50	94.9 ± 0.6	1.8 ± 0.2
B2-59	1220 ± 30	1670 ± 50	94.3 ± 0.4	2.7 ± 0.1
B2-83	980 ± 30	1710 ± 50	87.2 ± 0.4	7.5 ± 0.5
B2-98	700 ± 30	1320 ± 50	79.5 ± 0.8	7.1 ± 0.5
